# A novel oral nutritional supplement improves gait speed and mitochondrial functioning compared to standard care in older adults with (or at risk of) undernutrition: results from a randomized controlled trial

**DOI:** 10.18632/aging.202912

**Published:** 2021-04-02

**Authors:** Pol Grootswagers, Ellen Smeets, Antwi-Boasiako Oteng, Lisette de Groot

**Affiliations:** 1 Division of Human Nutrition and Health, Wageningen University, Wageningen, The Netherlands

**Keywords:** malnutrition, muscle, walking performance, ursolic acid, mitochondria

## Abstract

Undernutrition in older adults is mainly addressed by oral nutritional supplements, which do not affect physical functioning. In this study, we tested a novel oral nutritional supplement that included whey and casein protein, ursolic acid, free branch-chained amino acids and vitamin D against a standard supplement. We included older adults (>65y) with (or at risk of) undernutrition (n=82) and randomized them to 12 weeks of novel or standard supplement.

Both groups showed significant increases in body mass. No within or between-group differences in lean body mass were observed. Fat mass increased significantly more in the standard than the novel supplement group (time*treatment effect P=0.045). The novel supplement group showed a larger improvement in walking performance on distances of 4m (treatment x time interaction P=0.048) and 400m (treatment x time interaction P=0.038) than the standard treatment group. Gene sets related to mitochondrial functioning and oxidative phosphorylation were upregulated in the novel supplement group and downregulated in the standard supplement group.

We conclude that a 12-week intervention with the novel supplement improved walking performance both during short and long distance as compared to a standard supplement, which can largely be explained by increased mitochondrial functioning in the group receiving the novel supplement.

## INTRODUCTION

Undernutrition is a medical condition that is highly prevalent in older adults [1] and is often accompanied by a loss of skeletal muscle mass and impaired physical functioning [2]. Undernutrition is a prevalent comorbidity of medical conditions, including cancer [3], chronic obstructive pulmonary disease [4] and COVID-19 [5] and is associated with compromised survival outcomes [3].

Undernutrition is mainly addressed by oral nutritional supplements (ONS). These supplements are often energy and protein-dense and contain a wide range of macronutrients and micronutrients such that they form complete nutrition [6]. From pooled results of 62 studies, Milne et al. concluded that ONS show a small but consistent increase in body weight [7] when used during a period of 12 weeks. On the other hand, these authors concluded that ONS do not improve physical functioning and only slightly increase arm muscle circumference, which is a measure of muscle and fat tissue. Improving a patient's physical functioning via ONS would be very desirable, as compromised physical functioning greatly affects their quality of life [8, 9].

The absence of clear effects on muscle mass and physical functioning is not surprising given the current formulation of present-day supplements. Many ONS contain only casein protein, while whey protein has shown to be superior to casein protein in promoting skeletal muscle anabolism [10, 11]. This superiority of whey over casein is mainly accredited to its higher content of leucine, which by itself can increase muscle protein synthesis on the short term [12–14]. However, long-term interventions with only leucine supplementation have failed to show beneficial effects on muscle protein synthesis [15] or, indeed, physical functioning. An explanation for the lack of long-term benefits might be that administration of only leucine decreases plasma concentrations of valine and isoleucine, which makes their availability rate-limiting for muscle protein synthesis [16]. This issue might be circumvented by supplementing a mixture of valine, isoleucine and leucine (together forming the branched-chain amino acids, BCAAs). Recent human and mouse gene expression studies identified other compounds with promising physiological potential, including ursolic acid (UA) [17]. UA is a pentacyclic triterpenoid molecule that naturally occurs in a variety of fruits and herbs. Data from gene expression studies show that UA downregulates the muscle atrophy-inducing genes Muscle RING finger 1 (MuRF-1) and Atrogin-1, while it upregulates insulin-like growth factor 1 (IGF-1) signalling [17]. In a mouse model, a 5-week diet enriched with 0.27% UA resulted in increased quadriceps weight and grip strength compared to a control diet [17]. In another mouse study, 2-week diets with 80 and 240 mg/kg UA improved endurance capacity [18].

Recently, several investigators have used ONS products with altered compositions to address the issue of the poor efficacy of current products. However, while some promising results have been reported, these were either not yet proven to be superior over existing products in head-to-head studies or only effective in combination with exercise [19, 20]. While exercise has been shown to improve muscle mass and physiological performance, there is a clinical need for strategies that are feasible under various limiting conditions. Hence, we set out to test a novel whey-based ONS, high in leucine and vitamin D that previously was found to provide additional benefits [19, 20] and reinforced with UA that may affect muscle metabolism [17]. This novel ONS was tested in a calorically restricted mouse model and found to preserve muscle mass while affecting regulatory pathways [21]. In this study, we compared the effects of this novel supplement with a standard supplement on body composition, physical functioning, blood markers, and skeletal muscle gene and protein expression in older adults with (or at risk of) undernutrition without changing their activity pattern and without additional exercise.

## MATERIALS AND METHODS

### Study design

The ProMO-Study (PROtein supplementation in Malnourished Older adults) was a randomized, standard-care controlled, open-label trial conducted at Wageningen University, The Netherlands, between July 2016 and August 2017. The primary aim of the study was to assess the effect of 12-weeks supplementation with a novel supplement on lean body mass. The study has been performed in accordance with the 1964 Declaration of Helsinki ethical standards and was approved by the medical and ethical committee of Wageningen University and registered at clinicaltrials.gov under NCT02683720.

### Study population

Participants were recruited via dieticians, geriatric outpatient clinics of two hospitals (Rijnstate, Arnhem, The Netherlands and Gelderse Vallei, Ede, The Netherlands), the volunteer database of Wageningen University, and via advertisements in local and online media. Interested participants were, after obtaining informed consent, screened for eligibility during a home visit. We included participants aged 65 years and older, with present (risk of) undernutrition defined as a score below 12 on the Mini Nutritional Assessment Tool – short form (MNA-sf). Exclusion criteria were an expected life-expectancy of <12 months, mental state incompatible with proper study conduct, performing over 2 h /w of resistance exercise, unstable organ failure, impaired kidney function (estimated glomerular filtration rate (eGRF) <30 ml/min /1.73m^2^, measured at baseline), allergy or sensitivity to milk proteins, chronic corticosteroid use, use of diabetes medication including metformin, change in medication use in the previous three months, use of antibiotics in the previous two months, use of oral nutritional supplementation in the previous three months, and use of >21 alcohol units per week. Full medication records of all participants were enquired, and any changes in medications during the trial were documented.

### Sample size

Sample size was calculated by using G-Power (G-Power Version 3.1.9.2. Kiel, Germany). To find a 1.0 kg difference in lean body mass between novel and standard supplement after 12 weeks, with a standard deviation of 1.4 kg [22], we calculated that 64 participants provide a power of 80% at a two-sided alpha of 0.05. We assumed a drop-out rate of 20% and an after-baseline exclusion for impaired kidney function of 1.5%, leading to a total sample size of 82 participants.

### Randomization

Participants were randomized by an independent researcher using a SAS-program (SAS Institute, Cary, NC) in permuted blocks of 4 participants, in a 1:1 ratio to novel or standard supplement. The study was open-label: the supplements of the novel and the standard supplement group differed in appearance and taste, but all participants assumed that they were given a product with novel characteristics.

### Study products

The novel supplement group consumed two portions of the novel supplement (Vital01, VitalNext B.V., Wageningen, The Netherlands) per day, each portion consisting of 63 grams of powder to be dissolved in a liquid of choice. The standard supplement group consumed two 200 ml bottles of Nutridrink (Nutridrink, Nutricia Advanced Medical Nutrition, Danone, Hoofddorp, The Netherlands) per day. The daily doses of the novel and the standard supplement product contained equal amounts of energy, carbohydrates, fats and protein, but differed in the type of protein and the amount of BCAAs, vitamin D and UA (see Table 1 for the nutritional content). Participants were advised to consume products after breakfast and lunch, but deviation from this advice was allowed to maximize compliance. On measurement days, participants were not allowed to consume products prior to the university visit.

**Table 1:** The nutritional content of the study products per daily prescription (two portions).

	**Standard supplement**	**Novel supplement**
Energy (kcal)	600	586
Fat (g)	23	23
Carbohydrate (g)	74	65
Protein (g)	24	22
Of which casein (g)	24	11
Of which whey (g)	0	11
Free branched-chain amino acids (g)	0	7
Vitamin D3 (μg)	4.4	10.8
Ursolic acid (mg)	0	206

### Study visits

Participants visited the Human Nutrition Research Unit of Wageningen University on three occasions (at week 0, 6 and 12). Participants arrived in the morning after a light breakfast. Participants were free to consume a light breakfast of choice but were instructed to use exactly the same breakfast at the follow-up visits, so without consuming the supplements. At all three visits, body composition was assessed, and blood was collected. At all study visits, blood collection was always carried out before any other outcome measurements. At visit 1 and 3, additional measurements of physical function were performed, and vastus lateralis tissue was collected in qualified subjects (see below).

### Body composition

The primary outcome of this study was lean body mass, assessed via dual-energy x-ray absorptiometry (DXA, Lunar Prodigy Advance; GE Health Care, Madison, WI, USA). Fat mass and appendicular lean mass were additionally quantified via DXA. Phase angle and changes in intra- and extracellular water were measured by multi-frequency bioimpedance vector analysis (BIVA, SFB7, Impedimed Limited, Pinkeba, QLD, Australia). Height was measured to the nearest 0.1 cm, at baseline, using a stadiometer. Participants measured their body weight daily before breakfast at home, on commercial weighing scales (König HC-PS100N, NEDIS, 's Hertogenbosch, The Netherlands), to the nearest 0.1 kg. Bodyweight was also assessed during visits to the nearest 0.1 kg, with a calibrated weighing scale (ED-6-T; Berkel, Rotterdam, The Netherlands).

### Strength

Isometric knee extension and knee flexion strength (in Newton) were measured by handheld dynamometry (MicroFET2, HOGGAN Scientific LLC, Salt Lake City, UT, USA). Participants were seated on an examination table, with their knees flexed at a 90-degree angle. The examiner was seated against the wall for stability, provided standardized verbal encouragements and applied counterforce to the dynamometer. The dynamometer was placed just above the ankle joint, at the anterior side of the leg for extension measurements and the posterior side for flexion measurements. Participants were instructed to gradually increase their force within the first second after the standardized countdown ('3-2-1-GO'). After this second, the participants provided maximum voluntary force for up to 4 seconds. For extension and flexion, participants started with one familiarisation trial, followed by six repetitions alternating between legs. Peak dominant and non-dominant flexion and extension force were used for analysis.

Handgrip strength was assessed by hydraulic dynamometry (Jamar, Jackson, MI, USA) to the nearest kg while participants were seated on a chair without armrests, with their arms flexed at a 90-degree angle. Each hand was measured three times in an alternating fashion. The highest readings of dominant and non-dominant handgrip strength were used for analysis.

### Physical performance

Physical performance was assessed via the short physical performance battery (SPPB), which consist of (1) a 4-meter usual gait speed walking test, (2) a repeated chair rise test and (3) a balance test. On all three tests, a score between 0 and 4 was given following the original SPPB protocol [23]. The walking performance was assessed with the 400-meter walk test. Participants were instructed to walk 20 laps (20 m per lap, 400 m in total), at their usual gait speed, without using walking aids. Participants were allowed to take rest breaks if needed. Time (in seconds) to complete the 400 m was used for analysis.

### Blood collection

At all three visits, phlebotomists drew two 3 ml serum tubes, one 5 ml EDTA-containing tube and one 3 ml Li-Heparin tube. The blood in serum tubes was allowed to clot at room temperature for 30 minutes. All tubes were centrifuged to separate plasma or serum. EDTA-plasma was divided over four 0.5 ml tubes, which were stored at -80 degrees until analysis. Serum and Li-Heparin tubes were transported to an external laboratory (Hospital Gelderse Vallei, Ede, The Netherlands) for same-day analysis. Results of kidney function (estimated glomerular filtration rate, eGFR) were obtained on the same day to immediately exclude participants when eGFR dropped below <30 ml/min /1.73m^2^. Levels of vitamin D (by Liquid chromatography-mass spectrometry), IGF-1 (by Luminescence-enhanced immuno-enzymatic assay) and albumin (by bromocersol purple method) were measured at the laboratory of Hospital Gelderse Vallei.

At the first and last visit, additional blood was collected in 9 ml citrate tubes for immunological assessments. These tubes were transported to an external laboratory (Sanquin, Amsterdam, The Netherlands), where peripheral blood mononuclear cells (PBMCs) were isolated by Ficoll-Paque separation, washed and counted. PBMC composition (T-cells, B-cells, monocytes and natural killer (NK) cells) was assessed by flow cytometry. PBMCs were stimulated (in triplicate) with a mix of Tetanus Toxoid, Tubulin PPD and Candida Albicans to trigger a memory reaction of T-cells. Production of interferon-gamma (IFNγ) and interleukin-13 (IL-13) was measured in supernatants by enzyme-linked immunosorbent assays.

### Vastus lateralis tissue collection

Vastus lateralis tissue biopsies were taken under local anaesthesia, by using a Bergström needle, in a subgroup of *n=*36 participants who were willing to undergo a muscle biopsy and were not using anticoagulants. One part of the collected tissue was immediately rolled until dry, checked for quantity and type of tissue, and snap-frozen in liquid nitrogen for MicroArray analysis. A second part of the collected tissue was adhered to an object-glass with Tissue Tek (Sakura Tissue Tek, Alphen a/d Rijn, the Netherlands) and frozen in thawing isopentane for Western Blot-analysis. Samples were frozen at -80 degrees until analysis. In *n=*21 participants, the tissue collection was successful both at baseline and after 12 weeks. We used these samples for the microarray analysis. In *n*=15 of these *n*=21 participants, enough tissue was collected to perform Western Blot analyses to assess protein expression.

### RNA isolation and microarray processing

RNA was purified from muscle biopsies using TRIzol (Life Technologies, Bleiswijk, the Netherlands), followed by an additional round of purification with RNeasy Microkit columns (Qiagen, Venlo, the Netherlands). The RNA quality was assessed using RNA 6000 nanochips on the Agilent 2100 bioanalyzer (Agilent Technologies, Amstelveen, the Netherlands). Total RNA (100 ng) was labelled using an Affymetrix WT plus reagent kit and hybridized to whole-genome GeneChip Human Gene 2.1 ST arrays coding 25.088 genes and transcripts (Life Technologies, Bleiswijk, the Netherlands). Sample labelling, hybridization to chips and image scanning was performed according to the manufacturer's instructions.

### Microarray data analysis

Microarray analysis was performed using MADMAX pipeline for statistical analysis of microarray data [24]. Quality control was performed, and all arrays met our criteria. For further analysis, a custom annotation was used based on reorganized oligonucleotide probes, which combines all individual probes for a gene [25]. Expression values were calculated using the robust multichip average (RMA) method, which includes quantile normalization [26]. Significant differences in expression were assessed using paired Intensity-Based Moderated T-statistic (IBMT [27]). Gene set enrichment analysis was performed with ClusterProfiler [28], based on ranked gene lists of three contrasts: Δ gene expression week 0 to week 12 in the standard supplement group, Δ gene expression week 0 to week 12 in the novel supplement group, and the Δ of these Δs to assess between-group differences over time. Overlaps in gene sets were calculated via the same method used in Migliavacca et al.:

(OL = 2 × *c*/(*n* + *m*) × 100, where *n* and *m* are the sizes of the two gene sets and *c* is the genes in common) [29].

All microarray data are MIAME compliant and have been submitted to the Gene Expression Omnibus (identifier: GSE136395).

### Western blots

Proteins were isolated from the vastus lateralis tissue biopsies by homogenizing the biopsies in ice-cold RIPA Lysis, and Extraction Buffer (25 mm Tris-HCl, pH 7.6, 150 mm NaCl, 1% Nonidet P-40, and 0.1% SDS; Thermo Fisher Scientific, Rockford, Il, USA) supplemented with protease and phosphatase inhibitors (Roche Diagnostics, Almere, The Netherlands). The protein lysates were quantified using the bicinchoninic acid assay (ThermoFisher) according to the manufacturer's protocol. Protein lysates (12 μg of protein/well) were loaded and run on 8–16% Criterion gels (Bio-Rad, Veenendaal, The Netherlands), transferred onto PVDF membrane, blocked and probed with specific antibodies. Membranes were incubated overnight with primary antibodies at 1:1000 dilutions for AMPK, p-AMPK, TFAM (respectively #2603, #2535, #7495, Cell Signaling Technology, Danvers, MA, USA), and PGC1α (#PA5-72948, ThermoFisher Scientific), and were followed by secondary antibody incubation with anti-rabbit IgG HRP from goat (#AP187P, Merck, Darmstadt, Germany) at 1:2500 dilution. Blocking and the incubation of primary and secondary antibodies were all performed in TBS, pH 7.5, 0.1% Tween 20 (TBS-T), and 5% (w/v) skimmed milk. Before and after the secondary antibody incubation, membranes were washed three times for 10 min each with TBS-T. Quantification was performed with the ChemiDoc MP system (Bio-Rad) and Clarity ECL substrate (Bio-Rad). GAPDH served as a loading control.

### Dietary intake

Participants were instructed to keep their background diet unchanged during the study. Participants filled out a 2-day food record on the two days preceding the baseline visit and on the same two days before the final visit. Additional interviews were performed by trained dietitians, using models to estimate portion size, with a focus on food products high in energy, protein, calcium or vitamin D. Nutrient intake was calculated by using the Dutch food composition database [30].

### Physical activity

All participants were instructed to keep their level of physical activity equal during the study period. In a subset of *n*=27, physical activity was measured by accelerometry (ActiGraph GTX3, 2009, Pensacola, FL, USA). Participants wore the accelerometer on an elastic belt on the hip for 14 consecutive days during all waking hours, except during bathing or swimming. The accelerometers measured acceleration and deceleration in 3 spatial dimensions, which facilitates the calculation of vector magnitude. The epoch interval for the accelerometer was set at 60 seconds. ActiGraph version 6.13.3 (ActiGraph, Pensacola, FL USA) was used to initialize and synchronize the accelerometers. Participants who recorded less than 10 hours of wear time per day or had data for less than four days were excluded from the analyses (*n*=3). Non-wear time was defined as any period of consecutive zero-counts for a minimum of 20 minutes. Data were averaged and expressed in counts per minute (CPM).

### Compliance

Compliance was measured via intake calendars and empty package count. Empty packages were collected during three home visits during the study period. At these visits, also new study products were delivered, and any issues regarding protocol adherence were evaluated. Participants rated products weekly on taste, smell and satiety on a 100 mm visual analogue scale. Participants were asked to report all side effects and to record every adverse event in a provided diary. Adverse events were further discussed with an independent physician, who also assessed the probability of a relation with the study products.

### Statistical analysis

Data were analyzed by intention to treat, with α set at 5%. Normality was assessed via visual inspection of QQ-plots. To test for changes over time, we used linear mixed models with time, treatment and time*treatment interaction as fixed effects and a random intercept for subjects. Correlated errors within repeated measurements were modelled with an autoregressive covariance structure. All models were adjusted for gender and height, and models for knee extension and flexion also for the observer, as this measure is especially prone to inter-examiner bias [31]. Additional adjustment for age and BMI was performed if it improved Akaike's Information Criterion. Baseline characteristics are presented as crude mean ± SD for normally distributed variables and median (p25-p75) for skewed variables. For changes over time, adjusted means plus 95% confidence intervals are presented. We used Bonferroni adjustment to account for multiple posthoc comparisons. Changes in protein expression and immunological responses were assessed via nonparametric tests (sign test for paired comparisons and Wilcoxon-Mann-Whitney for comparisons between independent samples). All analyses were performed in SAS 9.4 (SAS Institute, Cary, NC), and graphs and figures were created using GraphPad Prism 5 (GraphPad Software Inc., San Diego, CA, USA).

## RESULTS

### Participants

**Figure 1:**
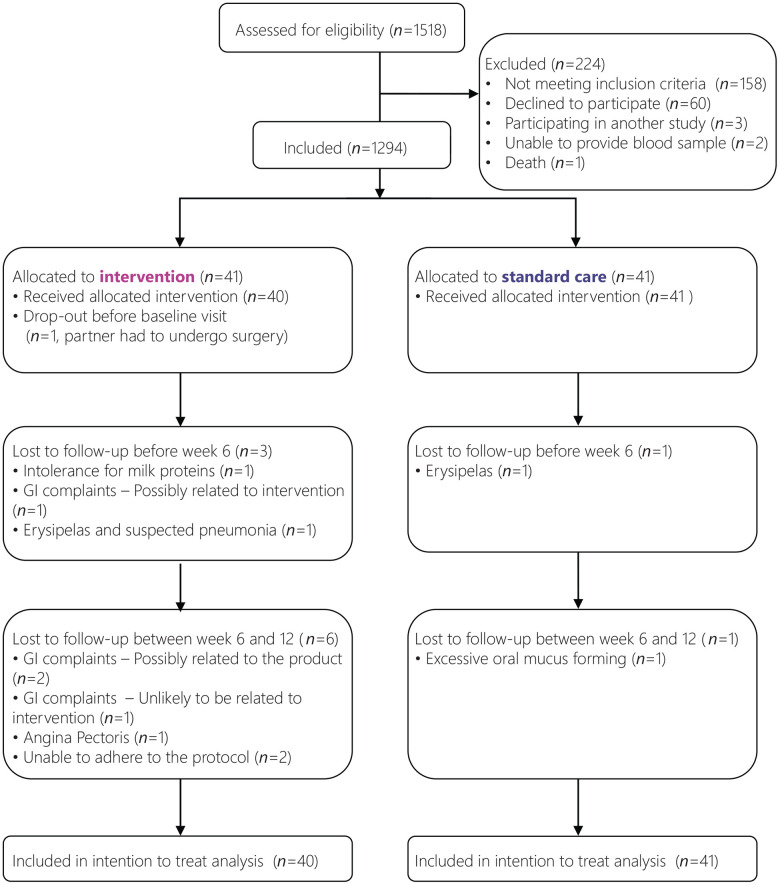
**Flowchart of participants.**

A total of 82 participants enrolled in the trial between August 2016 and May 2017, with the final measurement performed in August 2017. A total of 12 participants did not complete the full study period, of which one dropped-out before baseline measurements. Therefore, data of 81 participants were used for the modified intention to treat analyses (see Figure 1 for flow chart). Participant characteristics were comparable between the study groups (Table 2). The range of MNA-sf scores (5-11) and the median MNA-sf score (10) were the same in each group. Also, MNA-sf scores were comparable amongst all EWGSOP2 [32] sarcopenia classifications, except for severe sarcopenia. The five participants with severe sarcopenia were equally distributed over the two treatment arms (Supplementary Table 1).

**Table 2:** Baseline characteristics of the two study arms.

	**Novel** **supplement (n=40)**	**Standard supplement (n=41)**
Age, mean ± SD, y	74.5 ± 6.2	73.9 ± 6.8
Female, n (%)	22 (56%)	19 (44%)
Weight, mean ± SD, kg	61.6 ± 10.4	63.9 ± 10.8
Height, mean ± SD, cm	167 ± 9	171 ± 8
BMI, mean ± SD, kg/m^2^	22 ± 3	22 ± 3
MNA-sf, median (IQR), score	10 (9-11)	10 (9-11)
Undernutrition (<9), n (%)	3 (7.3%)	6 (14.6%)
Risk of undernutrition (8-11), n (%)	37 (92.7%)	35 (85.4%)
Phase Angle, mean ± SD, °	4.8 ± 0.8	4.7 ± 0.7
Total lean mass, mean ± SD, kg	45.0 ± 8.5	48.2 ± 9.1
Appendicular lean mass, mean ± SD, kg	18.8 ± 4.2	20.6 ± 4.7
Total fat mass, mean ± SD, kg	14.2 ± 6.4	13.1 ± 7.9
Protein intake, mean ± SD, g/kg/day	1.4 ± 0.4	1.3 ± 0.4
Energy intake, mean ± SD, kcal/day	2090 ± 452	2261 ± 548
Walk time 4m, mean ± SD, s	4.2 ± 0.9	4.4 ± 1.4
Walk time 400m, mean ± SD, s	355 ± 76	362 ± 102
Dominant handgrip strength, mean ± SD, kg	22 ± 10	23 ± 10
Non-dominant handgrip strength, mean ± SD, kg	20 ± 10	21 ± 10
Dominant knee flexion strength, mean ± SD, N	193 ± 70	188 ± 70
Non-dominant knee flexion strength, mean ± SD, N	184 ± 69	185 ± 73
Dominant knee extension strength, mean ± SD, N	330 ± 112	325 ± 94
Non-dominant knee extension strength, mean ± SD, N	312 ± 99	311 ± 97
Chair rise test time, mean ± SD, s	11.5 ± 4.9	10.8 ± 3.2
SPPB Score, median (IQR)	11 (10-12)	12 (10-12)

MNA-sf, Mini Nutritional Assessment tool, short-form; IGF-1, Insulin-like Growth Factor 1; SPPB, Short Physical Performance Battery.

### Body composition

**Figure 2:**
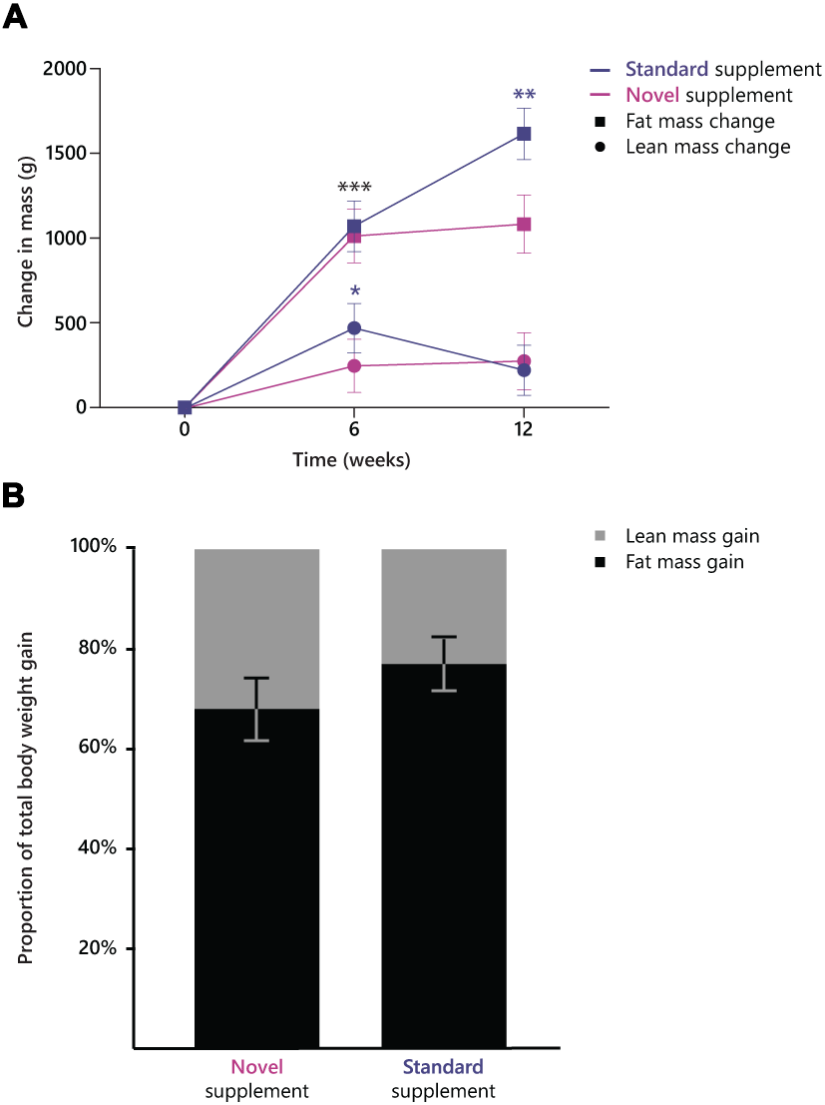
(**A**) Change in lean mass and fat mass over the course of the intervention period in the two treatment groups. ***, significant increase in fat mass within both groups between week 0 and 6; **, significant increase in fat mass within the standard supplement group between week 6 and 12; *, significant increase in lean mass between week 0 and 6 in the standard supplement group. (**B**) proportion of fat mass (79.5% in novel supplement, 87.7% in standard supplement) and (appendicular) lean mass (20.5% in novel supplement, 12.2% in standard supplement) as part of the total weight gain in the two groups.

Self-measured body weight increased in a similar manner in both treatment arms (P-value for treatment x time interaction >.05), in the novel supplement group from 61.6 kg (95% CI, 59.3-63.8) to 63.2 kg (95% CI, 60.9-65.4) and in the standard supplement group from 62.1 kg (95% CI, 59.9-64.3) to 63.8 kg (95% CI, 61.6-66.0). No within, or between-group differences in total lean body mass or appendicular lean body mass were observed (time*treatment effects P>0.05, Figure 2A). Fat mass increased differentially between the two treatment arms (time*treatment effect P=0.045, Figure 2). The standard supplement group showed a larger increase in fat mass (from 13.3 kg, 95% CI, 11.2-15.4 to 14.9 kg, 95% CI, 12.8-17.0) than the novel supplement group (from 13.9 kg, 95% CI, 11.7-16.1 to 15.0 kg, 95% CI, 12.8-17.2). The proportion of fat mass gain as part of total weight gain was 87.7 % in the standard supplement group and 79.5 % (Figure 2B). Phase angle tended to increase more in the standard supplement group (from 4.7°, 95% CI, 4.5-4.9 to 4.9°, 95% CI, 4.7-5.1) than in the novel supplement group (from 4.8°, 95% CI, 4.6-5.0 to 4.9°, 95% CI, 4.7-5.1, treatment x time interaction P=0.07).

### Physical function

**Figure 3:**
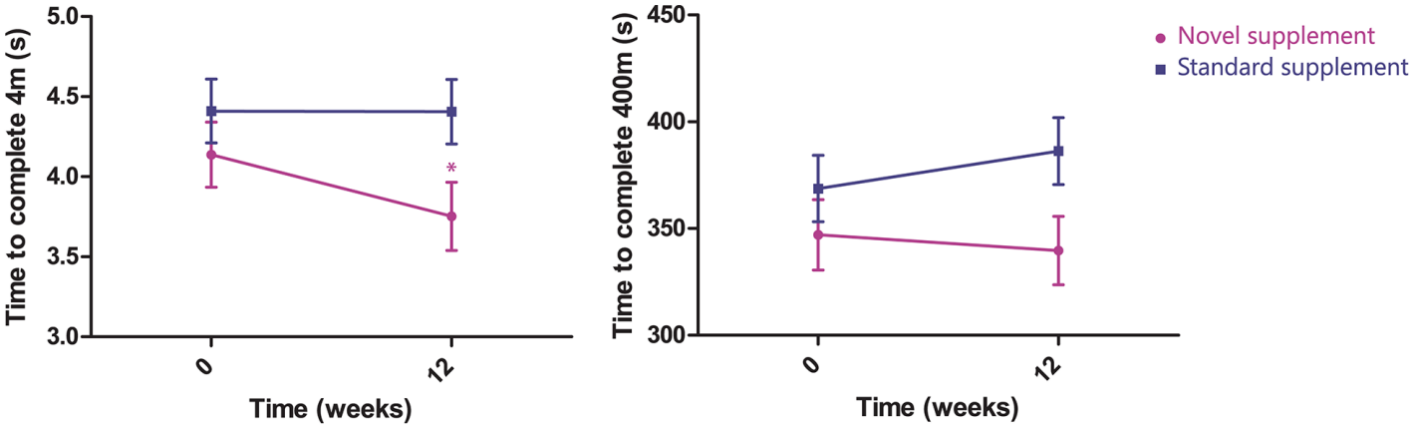
Change in time to complete 4 m (left, time*treatment effect P=0.047) and 400 m (right, time*treatment effect P=0.038, n=81).

The performance on the walk tests of 400 m and 4 m changed differentially over the two treatments arms during the study period in favour of the novel supplement group (400m, treatment x time interaction P=0.038; 4m, treatment x time interaction P=0.048; Figure 3). Time needed to complete the 400 m walk test changed from 347 s (95% CI, 316-378) to 340 s (95% CI, 308-372) in the novel supplement group, and from 369 s (95% CI, 338-400) to 386 s (95% CI, 355-417) in the standard supplement group. Time needed to complete the 4 m walk test changed in the novel supplement group from 4.1 s (95% CI, 3.7-4.5) to 3.8 s (95% CI, 3.4-4.2), and in the standard supplement group from 4.4 s (95% CI, 4.0-4.8) to 4.4 s (95% CI, 4.0-4.8).

Non-dominant knee extension increased in the standard supplement group from 296 N (95% CI, 266-325) to 334 N (95% CI, 305-363) and did not change in the novel supplement group (315 N, 95% CI, 288-342 to 322 N, 95% CI, 293-351) following the 12-week intervention (treatment x time interaction P=0.058). Dominant knee extension, and knee flexion of both legs, did not change within or between the treatment arms. Moreover, no improvements were observed in handgrip strength, chair rise test, or total SPPB score for both groups (Table 3). Activation of vastus lateralis, biceps femoris and rectus femoris as measured by surface electromyography did not differ within or between groups.

**Table 3:** Changes in physical function in the two groups.

		**Δ12 weeks**				**Time effect**	Time*treatment interaction
		**Novel supplement (n=40)**	**P-value**	**Standard supplement** **(n=41)**	**P-value^a^**		
Walking performance	4 m^b^	-0.4 ± 0.1 s	0.047	0.0 ± 0.1 s	1.000	0.044	0.048
	400 m^c^	-7.4 ± 8.7 s	1.000	17.6 ± 7.8 s	0.172	0.386	0.038
Handgrip Strength^b^	Dom	0 ± 1 kg	1.000	0 ± 1 kg	1.000	0.974	0.948
	Non-dom	0 ± 1 kg	1.000	1 ± 1 kg	1.000	0.660	0.340
Knee extension^d^	Dom	-2 ± 14 N	1.000	26 ± 21 N	1.000	0.200	0.145
	Non-dom	8 ± 12 N	1.000	38 ± 10 N	0.003	0.005	0.058
Knee flexion^d^	Dom	12 ± 9 N	1.000	23 ± 8 N	0.036	0.006	0.351
	Non-dom	6 ± 10 N	1.000	15 ± 8 N	0.443	0.106	0.453
Chair rise test^c^		0.0 ± 0.5 s	1.000	-0.3 ± 0.4 s	1.000	0.543	0.634
SPPB score		0.1 ± 0.2 pt	1.000	0.3 ± 0.2 pt	0.523	0.169	0.355

Dom, dominant limb; non-dom, non-dominant limb; N, Newton; SPPB, short physical performance battery.

^a^P-values are from posthoc tests with Bonferroni adjustment of multiple testing.

^b^Adjusted for gender and height.

^c^Adjusted for gender, height and age.

^d^Adjusted for gender, height, age and observer.

### mRNA expression

**Figure 4:**
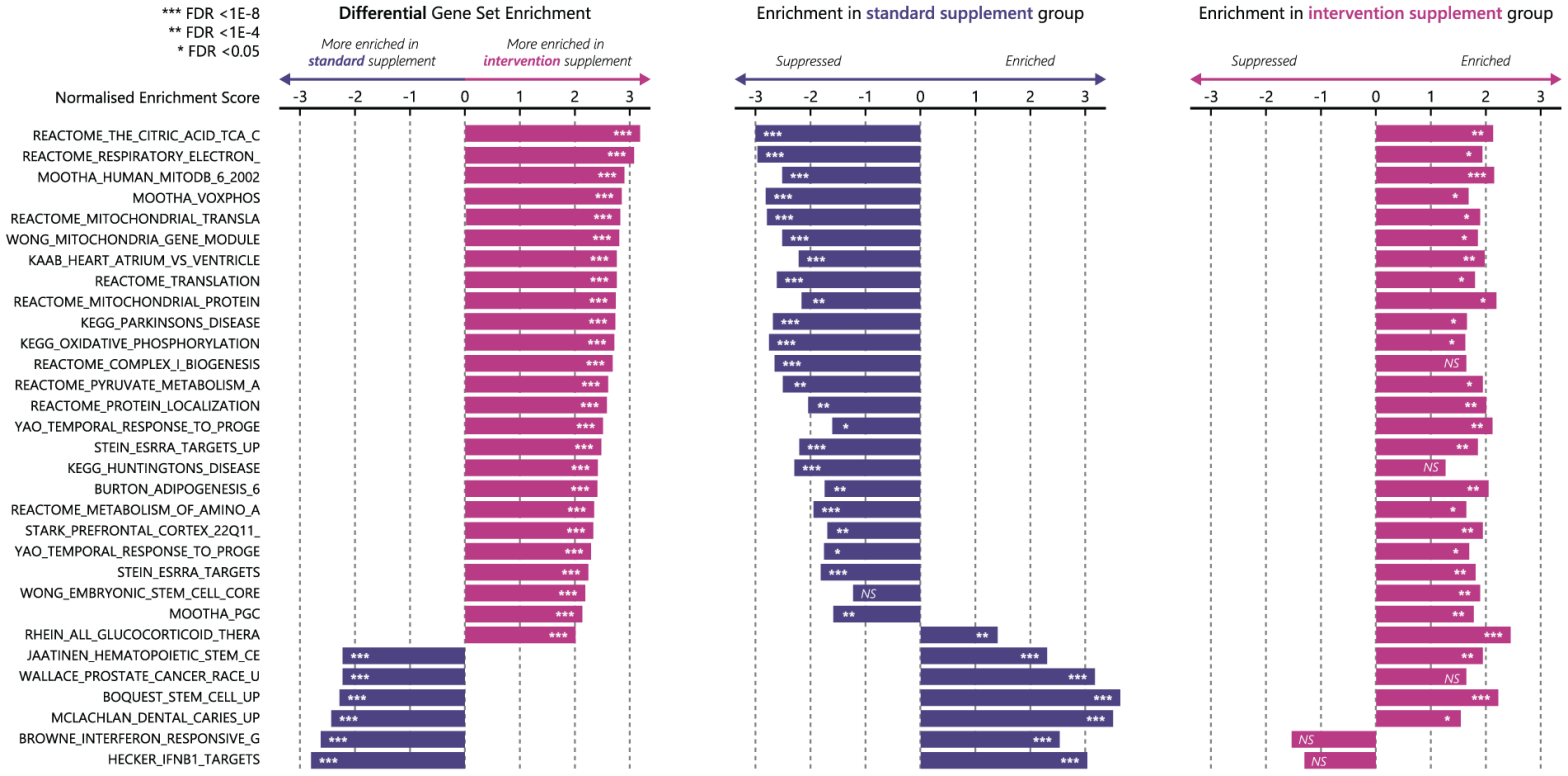
**Enrichment of the top enriched gene sets (FDR<1.5 E-8, overlap < 75%) from differential analysis in *ClusterProfiler* using MsigDB C2 Curated gene sets.** From left to right, differential enrichment of gene sets, enrichment of these gene sets in standard supplement group (*n=*13, blue bars), and enrichment of these gene sets in novel supplement group (*n=*7, pink bars). FDR, false detection rate; NS, not significant.

**Figure 5:**
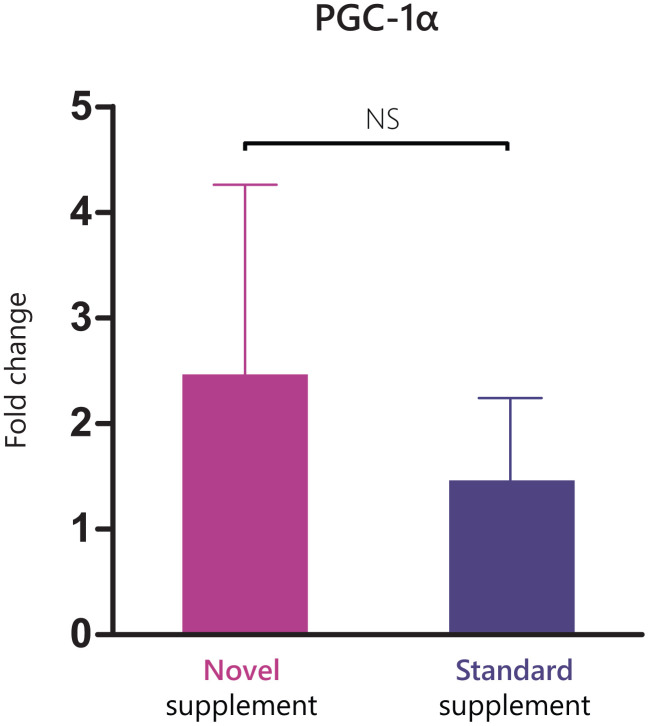
**Fold change in PGC1-α expression between baseline and week 12 in the two treatment arms.**

The gene sets that were differentially enriched with an FDR<1.5E-8 and overlap of <75% are presented in Figure 4. The largest between-group differences in gene set enrichment between week 0 and week 12 were observed in pathways related to oxidative phosphorylation, mitochondrial functioning, and mitochondrial biogenesis out of all C2 curated gene sets that were tested. Results from Western Blot analyses on protein expression are presented in Supplementary Materials and in Supplementary Figure 1. The fold change expression of PGC1-α was 4.7 ± 1.8 in the novel supplement group and 2.2 ± 0.6 in the standard supplement group, with no significant between treatment differences (P=0.685, Figure 5).

### Dietary intake and physical activity

Daily energy intake at baseline was 2090 ± 452 kcal in the novel supplement group and 2261 ± 548 kcal in the standard supplement group. Energy intake increased by 409 ± 95 kcal/d in the novel supplement group (P<0.001) and by 284 ± 87 kcal/d in the standard supplement group (P=0.01). Protein intake increased in both groups: in the novel supplement group from 83.4 ± 4.3 g/d to 102.8 ± 4.7 g/d (Δ 19.4 ± 4.4 g/d, P<0.001) and in the standard supplement group from 83.8 ± 4.3 g/d to 100.7 ± 4.3 g/d (Δ 16.9 ± 4.0 g/d, P<0.001).

Physical activity was equal between groups at baseline (Δ 138 ± 74 CPM, P=0.498) and end of follow-up (Δ 124 ± 88 CPM, P=1.000). No change in physical activity was observed in the novel supplement group (Δ -33 ± 62 CPM, P=1.000) and the standard supplement group (Δ -19 ± 47 CPM, P=1.000).

### Blood markers

The pre- and post-treatment values of blood markers and PBMC composition are presented in Table 4. Mean baseline vitamin D levels were 82.7 ± 4.2 nmol/l in the novel supplement group and 77.0 ± 4.1 nmol/l in the standard supplement group (Δ 5.7 ± 5.9 nmol/l, P=1.000). Both groups showed similar, highly significant increases in vitamin D status throughout the study period. Status levels increased on average with 13.3 ± 3.0 nmol/l (P<0.001) in the novel supplement group and with 12.7 ± 2.7 nmol/l (P<0.001) in the standard supplement group. IGF-1 levels were similar between groups at baseline (19.2 ± 0.9 nmol/l vs 16.2 ± 0.9 nmol/l, Δ 3.0 ± 1.3 nmol/l, P>0.05). IGF-1 levels decreased in the novel supplement group (Δ -0.9 ± 0.6 nmol/l, P>0.05), while they increased in the standard supplement group (Δ 1.3 ± 0.6 nmol/l, P>0.05), resulting in a significant time*treatment effect (P=0.025).

**Table 4:** Changes in blood markers and PBMC composition.

	**Novel supplement (n=40)**			**Standard supplement (n=41)**		Time* treatment interaction
	**PRE** **treatment**	**POST treatment**		**PRE treatment**	**POST treatment**	
Albumin (g/l)	38.9 ± 0.4	36.6 ± 0.4***		38.4 ± 0.4	37.3 ± 0.4	0.002
Creatinine (μmol/l)	77.0 ± 2.8	73.8 ± 2.9		78.4 ± 2.8	79.3 ± 2.8	0.004
IGF-1 (nmol/l)	19.2 ± 0.9	18.3 ± 1.0		16.2 ± 0.9	17.5 ± 0.9	0.025
25(OH)D (nmol/l)	82.7 ± 4.2	96.0 ± 4.4**		77.0 ± 4.1	89.6 ± 4.2***	NS
T-cells (%)	56 ± 12	61 ± 12*		61 ± 11	63 ± 10*	NA
NK-cells (%)	17 ± 9	15 ± 8		17 ± 8	15 ± 7**	NA
B-cells (%)	5.9 ± 2.3	6.4 ± 2.6		5 ± 2	5 ± 2	NA
Monocytes (%)	16 ± 8	12 ± 5*		13 ± 8	13 ± 6	NA

PBMC, peripheral blood mononuclear cell; NK, natural killer. *, P<0.05; **, P<0.01; ***, P<0.001.

Blood creatinine levels decreased from 77.0 ± 2.8 μmol/l to 73.8 ± 2.9 μmol/l in the novel supplement group, while the standard supplement group showed a minor increase from 78.4 ± 2.8 μmol/l to 79.3 ± 2.8 μmol/l (time*treatment interaction P=0.004). Albumin decreased in the standard supplement group with 1.0 ± 0.4 g/l (P=0.08) and in the novel supplement group with 2.2 ± 0.4 g/l (P<.0001).

### Immune function

Experiments on PBMCs were performed in *n*= 69 participants who completed the before and after blood collection. The composition of the PBMCs changed in both groups. In the standard supplement group, a slight increase in %T-cells (61 ± 11% to 63 ± 10%, P<0.05) was observed, at the expense of a decrease in %NK-cells (17 ± 8% to 15 ± 7%, P<0.001). The novel supplement group showed a large increase in %T-cells (56 ± 12% to 61 ± 12%, P<0.01) and also in %B-cells (5.9 ± 2.3% to 6.4 ± 2.6%, P=0.064), whereas decreases in %monocytes (16 ± 8% to 12 ± 5%, P<0.01) and %NK-cells (17 ± 9% to 15 ± 8%, P=0.08) were observed. In both groups, there were no differences in before-stimulation levels of IL-13 and IFNγ at baseline compared to end-of follow-up. Stimulation of PBMCs with memory recall mixes significantly increased levels of IL-13 and IFNγ in both groups at both time points (P<.0001 for all comparisons). Before and after stimulation levels of IL-13 and IFNγ did not differ between groups on both time points.

### Tolerance

Median compliance was high (95%) and did not differ between treatment arms. The taste of the standard supplement product was rated better on a 100 mm VAS scale than that of the novel supplement at the start of the trial (standard supplement: 57 ± 3.6 mm vs novel supplement: 40 ± 3.6 mm, P=0.001). This difference attenuated during the trial, resulting in a tendency to a higher rating for the standard supplement product at the end of follow-up (standard supplement: 60 ± 4.7 mm vs novel supplement: 49 ± 4.7 mm, P=0.085).

During follow-up, a total of 4 serious adverse events occurred, which were not related to treatment nor to study procedures. All serious adverse events (angina pectoris, erysipelas with suspected pneumonia, transient ischemic attack, volvulus) occurred in the novel supplement group, and the first two led to discontinuation of the participant. Adverse events were reported by 31 (77.5%) participants in the novel supplement group and 28 (68.3%) participants in the standard supplement group. A total of 55 adverse events were classified as gastro-intestinal complaints, which occurred in 42 participants (Table 5). Upper abdominal complaints, such as belching, heartburn and dyspepsia, were reported more often by participants in the standard supplement group (n=10, leading to drop-out in n=1) than in the novel supplement group (n=5). Side-effects related to defecation, mainly reported as diarrhoea, were observed more often in participants in the novel supplement group (n=21) than in the standard supplement group (n=7). In the novel supplement group, these complaints led to study discontinuation in 5 participants. The relation to the study product was evaluated by an independent physician and considered 'definite' in n=1 (intolerance for milk proteins which was not reported by the participant during screening), 'possible' in n=3 and 'unlikely' in n=1 of the participants who dropped out.

**Table 5:** Overview of gastro-intestinal adverse events that occurred during the trial.

**Adverse event**	**Novel supplement (n=40)**	**Standard supplement (n=41)**
Participants with any reported event (n)	31	28
Of which gastro-intestinal (n)	25	17
Belching, heartburn, dyspepsia (n)	5	10
Altered defecation, such as constipation or diarrhoea (n)	21	7
Gastro-intestinal complaints leading to drop-out (n)	5	1
Of which relation to study product was independently evaluated as:		
Definite (n)	1	0
Possible (n)	3	1
Unlikely (n)	1	0

## DISCUSSION

In this study, twelve weeks of supplementation with a novel supplement containing a mixture of casein and whey protein, additional free branched-chain amino acids, additional vitamin D, and ursolic acid achieved a significant increase in body weight, which was comparable to standard supplement. Participants receiving standard supplement gained significantly more fat mass compared to those receiving the novel supplement. The relatively larger gain in lean mass in participants receiving the novel supplement did not lead to significant between-group differences. In the total sample, lean mass did increase significantly, even in the absence of concurrent resistance exercise. Treatment with the novel supplement led to an improved gait speed over short and long distance. Transcriptomics on muscle tissue suggest that improved mitochondrial functioning may have led to the observed improvements in muscle functioning.

The significant increase in lean mass in the total population amounted to 0.25 kg, of which 0.17 kg was appendicular lean mass, ALM. The 0.17 kg increase in ALM was in line with findings from the PROVIDE study, which found a 0.17 kg higher ALM after 13 weeks of supplementation with a whey protein-based ONS compared to non-protein containing control in older adults suffering from sarcopenia [20]. In our study, the standard supplement led to a considerably greater increase in fat mass than the novel supplement did (1.6 vs 1.1 kg), while no significant between-group differences in body weight gain were observed. Consequently, the relative amount of lean mass as part of the total gained mass was higher in the novel supplement group (21%) than in the standard supplement group (12%). The lower fat mass gain in the novel supplement group might be due to anti-obesity effects of UA that have been reported in multiple animal studies [33] and a human trial [34]. It is unlikely that the different types of proteins in the supplements caused this difference [35]. The standard supplement group also showed a greater improvement in phase angle than the novel supplement group. Phase angle is considered as a measure for undernutrition, but its diagnostic value is debatable [36].

Participants in the novel supplement group improved their walking performance on the 4m and the 400m walk test, while participants in the standard supplement group showed a stable (4m walk test) or even decreased walking performance (400m walk test). The improvements in gait speed that we observed are clinically relevant [37, 38] and might lower the chance of falls, hospitalization and mortality [39, 40]. The effect on gait speed is likely explained by a difference in mitochondrial functioning. Our mRNA expression analyses showed that of all C2 curated gene sets, gene sets related to oxidative phosphorylation, mitochondrial dynamics and mitochondrial biogenesis were most differentially expressed between the two groups. These gene sets were enriched in participants receiving the novel supplement, while they were strongly suppressed in participants receiving the standard supplement. Thus, participants receiving the novel supplement showed enrichments in mitochondrial gene sets and improvements in gait speed, while participants receiving standard supplement showed suppressions of the same gene sets and declines in gait speed.

The similar pattern between mitochondrial dynamics and performance on gait speed is not surprising. Studies have shown that lower oxidative phosphorylation capacity is related to higher fatigability in older adults [41] and that the mitochondrial capacity for oxidative phosphorylation correlates well with walking speed in studies with comparable populations [42, 43]. Importantly, participants in both groups of the present study did not increase their physical activity level during the trial period, a prominent intermediate in the relation between mitochondrial energetics and physical functioning [44]. Recently, Migliavacca et al. showed that sarcopenia is related to pronounced downregulation of several mitochondrial gene sets [29]. Strikingly, the participants who received the novel supplement in our study showed enrichments in exactly the same gene sets, indicating a sarcopenia-opposing epigenetic fingerprint induced by the novel supplement.

The most likely compounds in the novel supplement to be responsible for the improvements in gait speed and oxidative metabolism are the free BCAAs and UA. BCAAs have shown to enhance mitochondrial biogenesis and endurance capacity in mouse models, which is mainly explained by an upregulation of mTOR [45, 46]. In our study, we did not observe upregulation of the mTOR pathway in either treatment arm. However, BCAAs also upregulate PGC-1α and SIRT-1 [45]. We also observed an upregulation of protein expression of PGC-1α, but we were not able to detect SIRT-1 proteins with western blots. Moreover, BCAAs are precursors of acetyl-CoA and succinyl-CoA, which are components of the TCA-cycle, and might, therefore, lead to upregulations of the TCA-cycle gene set.

Of interest, Chen and colleagues showed that UA improved mitochondrial biogenesis via upregulation of activated AMPK, TFAM and PGC-1α in myotubes, and improved endurance in mice [18]. In the subsample of *n*=15 participants from which we successfully assessed protein expression via western blots, we found results that are in line with these findings. Whereas we observed increased activation of AMPK in both treatment arms, the indications of increased expression of PGC-1α was especially pronounced in the novel supplement group. TFAM was also upregulated in both treatment arms, but in this case, the expression was increased to a greater extent in the standard supplement group. Within-group upregulations of TFAM and PGC-1α were, however, not significant due to large variation. All in all, the potential of UA and of the intervention supplement on mitochondrial dynamics deserve attention in the future, as improving mitochondrial functioning might prove pivotal for the muscle functioning of older adults [47].

Our study design does not allow us to determine the compound that is responsible for the observed effects of the novel supplement. However, our results on endurance and mitochondrial protein expression are strikingly similar to the results found with isolated UA on muscle fibres and earlier in mouse studies. Therefore, we do urge the need for highly controlled trials that investigate the role of isolated UA on endurance capacity and mitochondrial functioning in older adults to further explore the potential therapeutic role of UA in this population.

We did not observe treatment-specific differences in muscle strength. Handgrip strength, total SPPB score and chair rise test did not show any sign of a change in either group. Extension and flexion of the legs increased in both groups, but only the extension of the non-dominant leg showed between-treatment differences, with a trend towards a beneficial effect of the standard supplement over the novel supplement group. However, the increase in non-dominant flexion in the standard supplement group failed to reach a significant time*treatment effect and did not translate into improved performance on chair rise test or the two walking tests.

We did observe an overall increase in lean mass during the intervention, indicating that a nutritional intervention without a concurrent (resistance) exercise program is able to improve lean body mass. This has important implications for clinical practice where exercise interventions are not always possible. Regardless, it would be of interest to further research the implications of the novel supplement with a concurrent exercise intervention, as it might largely augment findings on lean mass and mitochondrial functioning.

We observed a time*treatment effect on IGF-1 levels, with an increase of 1.3 nmol/l in the standard supplement group and a decrease of -0.9 nmol/l in the novel supplement group. As IGF-1 levels were 3.0 nmol/l higher in the novel supplement group at baseline compared to the standard supplement group, this observed effect could be partly explained by regression to the mean. Moreover, there is evidence showing that only casein protein, and not whey, increases IGF-1 levels [48]. These different effects of whey and casein on IGF-1 levels possibly explain why the standard supplement product, which contained 24 g of casein per daily dose, resulted in increased IGF-1 levels, while the intervention product, which contained 11 g of casein and 11 g of whey per daily dose, did not increase IGF-1 levels. Moreover, although UA stimulated IGF-1 signalling in skeletal muscle tissue, it did not increase plasma IGF-1 levels in mouse models [17]. The exact effects of plasma IGF-1, which is primarily produced hepatically under the influence of growth hormone, on lean body mass in older adults are unclear [49, 50]. Also, in our study, the between-group differences in plasma IGF-1 levels did not translate into differences in lean body mass.

In both groups, participants showed high compliance to the study products and rated both products equally well, suggesting that nutrition supplementation in fluid or powder form is both acceptable for patients. Thereby, the supplements successfully increased protein and energy intake. Participants in this study already had a high mean protein intake (1.4 g/kg/day), energy intake (2177 kcal/day) and vitamin D status (80 nmol/l) at baseline. These high intake levels might be explained by increased intake or over-reporting, as a result of increased awareness towards food intake. A cause for this could be the nutritional assessment (by MNA-sf) performed at the screening visit, before the days on which the baseline food records were completed, or the fact that some participants were already seeing a dietitian, and dietary advice other than ONS may already have been implemented (for instance consuming more dairy). Although the daily vitamin D intake from ONS was more than twice as high in the novel supplement group (10.8 μg) than that in the standard supplement group (4.4 μg), both groups showed a similar increase in vitamin D status. This can be explained by the high baseline vitamin D levels, variations in vitamin D intake from the background diet and the allowed use of vitamin D supplementation during the study. Despite the compliance with the nutritional therapy and the improvements in body weight, serum albumin levels decreased in both treatment arms. This observation supports the growing consensus that albumin levels are not suitable as a marker for nutritional status, as many other factors influence serum albumin levels [51]. Results of the stimulation of PBMCs with memory recall mixes did not suggest any substantial changes in immune function on both groups, although the increased proportion of T and B cells in the novel supplement group may impact the general immune status of the subjects. Of note, the same novel supplement seemed to increase the expression of immune-protective pathways in mice [21].

The study reported in this paper is one of the larger randomized controlled trials with ONS run in community-dwelling participants with (risk of) undernutrition. Trials with ONS in undernourished older adults are scarce, are often lacking assessment of important outcomes such as physical functioning and body composition, and often do not have a suitable comparator therapy [52]. We compared the novel supplement to standard care, which allows examination of its real added value, in the absence of exercise, which reflects the real-life situation of community-dwelling older adults. The broad range of measured outcomes provides an extensive understanding of the effects of the novel supplement in comparison to the standard supplement product. Notably, the inclusion of muscle mRNA and protein expression analyses provided crucial information on underlying mechanisms. We observed higher median compliance (95%) compared to other studies that investigated ONS in community settings (81%) [53]. Moreover, while we anticipated a drop-out rate of 20%, only 13.5% of participants did not complete the study due to a variety of reasons. In the standard supplement group, drop-out rates were low, with only 2 of 41 participants dropping out. The drop-out rate was higher in the novel supplement group, partly due to gastro-intestinal complaints possibly related to the study product in 4 of 40 participants. One of these participants appeared to be intolerant to milk protein that had not been reported. For the other three, we did not find an explanation for their reaction to the product, and an independent physician evaluated these complaints as being possibly related to the study product.

This study also faced some limitations. First, due to the different appearance of the study products, blinding was not possible. However, participants did not know which product was the intervention product and which was the control product. In this way, all participants were under the impression that they received the product of interest, which diminished a placebo effect in the novel supplement group. Besides, several objective outcomes (such as DXA and mRNA expression) are not prone to caveats of a non-blinded design. For rated outcomes, examiners were extensively trained on following standardized protocols to avoid influencing results. Second, the multiple differences between study products make it impossible to identify with certainty which compound is responsible for the found effects. Third, we advised participants to consume the products after breakfast and lunch, but they were free to follow this advice or to distribute the supplements over the day in their preferred way. As a result, some participants may not have reached the anabolic threshold during every meal, but this did contribute to high compliance and mimicked the real-life situation. Finally, this study included community-dwelling participants with no clear cognitive or physical impairments. For practical relevance, including such highly vulnerable populations would be much welcomed. Possibly, greater improvements in physical functioning could be observed in case participants have a lower physical functioning at baseline and participants with cognitive dysfunction might particularly benefit from improvements in mitochondrial functioning [54]. On the other hand, in those with cognitive impairments, the compliance to the supplements could be lower, especially to the novel supplement that needs to be dissolved in a liquid.

In conclusion, we showed that 12-week supplementation with a novel oral nutritional supplement improved walking performance in older adults with (or at risk of) undernutrition, possibly via improvements in mitochondrial mechanisms in the muscle. Compared to standard care, the novel supplement performed equally well on other measured domains of physical function and body composition.

### Data-share statement

Data described in the manuscript, codebook, and analytic code will be made available upon request.

## Supplementary Material

Supplementary Materials
