# Supplementary material for: A novel oral nutritional supplement improves gait speed and mitochondrial functioning compared to standard care in older adults with (or at risk of) undernutrition: results from a randomized controlled trial
Source: Aging (Albany NY). 2021 Apr 2;13(7):9398–418. doi: 10.18632/aging.202912 (PMC8064187; doi:10.18632/aging.202912)
Supplement: Supplementary Materials [file aging-13-202912-s001.pdf]

SUPPLEMENTARY MATERIALS

MNA-score per sarcopenia classification

Supplementary Table 1. Mean MNA-score per EWGSOP2 (Cruz-Jentoft et al., 2019) sarcopenia classification.

	Mean MNA-score	SD	N-total	N Novel supplement arm	N Standard supplement arm
No Sarcopenia	9.89	1.306	47	25	22
Sarcopenia Probable	9.22	1.649	27	12	15
Sarcopenia Confirmed	9.67	1.155	3	1	2
Sarcopenia Severe	8.60	2.302	5	3	2
Total	9.59	1.515	82	41	41

Protein expression

Both treatments upregulated the expression of proteins that induce mitochondrial biogenesis (see Figure). Activation of AMPK increased significantly in the novel supplement group (P=0.031) compared to the standard supplement group (P=0.125) (Figure 4B). TFAM and PGC-1α expressions were not differentially upregulated between the two groups (Figures 4C, 4D, P=0.603 and P=0.685 for between treatment differences in TFAM and PGC-1α fold changes, respectively).

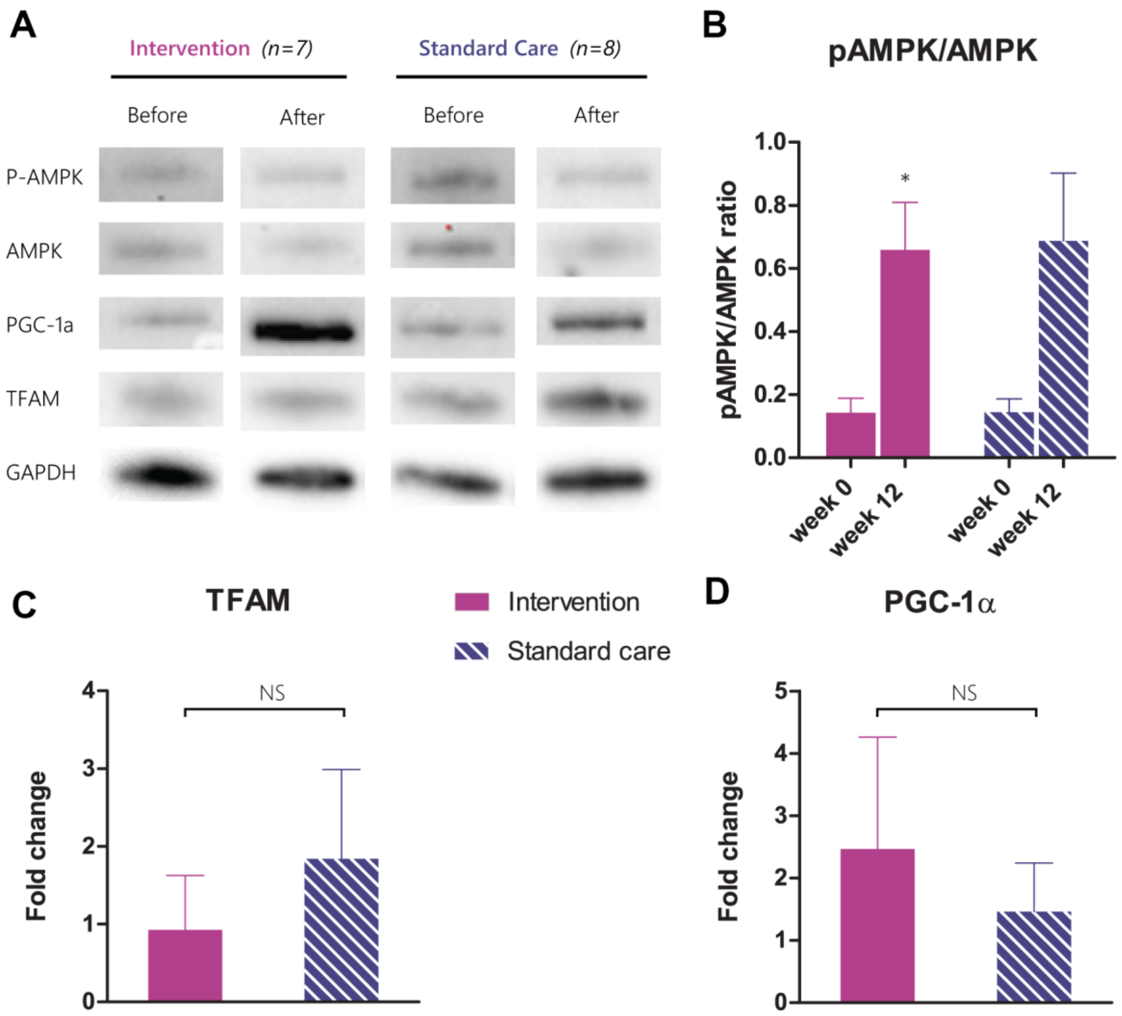

Supplementary Figure 1. Effects of the two treatments on expression levels of proteins involved in mitochondrial pathways (n=15). Representative captures from western blots (**A**), of which quantification (mean plus standard error bar) is presented in (**B**–**D**): pAMPK/AMPK ratios per treatment arm and per measurement time (**B**), fold change between week 0 and 12 in arbitrary units of TFAM (**C**) and PGC-1α (**D**) expression. All protein densities were normalised against GAPDH expression. *, significant (P<.05) within-group change from baseline to week 12.
